# Measures for concordance and discordance with applications in disease control and prevention

**DOI:** 10.1177/0962280218796252

**Published:** 2018-09-03

**Authors:** Marc Aerts, Adelino JC Juga, Niel Hens

**Affiliations:** 1 Interuniversity Institute for Biostatistics and statistical Bioinformatics, Hasselt University, Diepenbeek, Belgium; 2 Department of Mathematics and Informatics, Faculty of Sciences, Eduardo Mondlane University, Maputo, Mozambique; 3 Centre for Health Economics Research and Modeling Infectious Diseases, Centre for the Evaluation of Vaccination, Vaccine & Infectious Disease Institute (WHO Collaborating Centre), University of Antwerp, Antwerp, Belgium

**Keywords:** Association, asynchrony, concordance, discordance, marginal homogeneity, maximum likelihood, McNemar’s test, synchrony

## Abstract

Bivariate binary response data appear in many applications. Interest goes most often to a parameterization of the joint probabilities in terms of the marginal success probabilities in combination with a measure for association, most often being the odds ratio. Using, for example, the bivariate Dale model, these parameters can be modelled as function of covariates. But the odds ratio and other measures for association are not always measuring the (joint) characteristic of interest. Agreement, concordance, and synchrony are in general facets of the joint distribution distinct from association, and the odds ratio as in the bivariate Dale model can be replaced by such an alternative measure. Here, we focus on the so-called conditional synchrony measure. But, as indicated by several authors, such a switch of parameter might lead to a parameterization that does not always lead to a permissible joint bivariate distribution. In this contribution, we propose a new parameterization in which the marginal success probabilities are replaced by other conditional probabilities as well. The new parameters, one homogeneity parameter and two synchrony/discordance parameters, guarantee that the joint distribution is always permissible. Moreover, having a very natural interpretation, they are of interest on their own. The applicability and interpretation of the new parameterization is shown for three interesting settings: quantifying HIV serodiscordance among couples in Mozambique, concordance in the infection status of two related viruses, and the diagnostic performance of an index test in the field of major depression disorders.

## 1 Introduction

In medical applications as well as in other fields, it is often of interest to examine the “resemblance” between two or more observations from paired or matched outcomes. Here the focus is on two binary paired or matched outcomes. Examples considered in this paper are the HIV status among couples; the infection statuses for the same individual for both the Varicella-Zoster Virus and the Parvo B19-virus, viruses that are similar in their transmission being close contact; and the diagnostic performance of the Whooley questions as a screening tool for depression amongst older adults in primary care. Resemblance can be measured in different ways, depending on the characteristic of interest. It could be represented by an association parameter, such as the Pearson product-moment correlation or, for binary data, by the cross-product ratio or odds ratio. However, often association is not of interest but rather agreement. Agreement and association are in general distinct facets of the joint distribution. Strong agreement requires strong association, but strong association can exist without strong agreement.^1^ A well-known measure for agreement is Cohen’s kappa, see Agresti^1^ for extensions and ways to model agreement.

Measures for association and agreement are typically symmetric and can be misleading if one of the agreeing outcomes is very dominant, such as the (negative, negative) combination in our first example of the HIV status among couples. Indeed, as the majority of pairs agree in being negative, symmetric measures of association or agreement might be high even if there is only a small number of agreeing positive pairs. In the context of measuring synchrony in neuronal firing, Faes et al.^2^ proposed a new measure of synchrony, the conditional synchrony measure (CSM), which is the probability of two neurons firing together, given that at least one of the two is active. Faes et al. state that, although the odds ratio is an attractive association measure with nice mathematical properties (such as the absence of range restrictions, regardless of the marginal probabilities), it is less suitable for quantifying synchrony, due to its symmetry treating 0–0 matches of equal importance as 1–1 matches. Similar to the CSM but being rather interested in discordance, Juga et al.^3^ defined the HIV conditional (sero)discordance measure (CDM) as the conditional probability that the couple is HIV discordant, given that at least one of them, man or woman, is HIV positive.

As noted by Faes et al. and Juga et al., a reparameterization of the joint bivariate binary distribution in terms of the marginal “success” probabilities and with the OR parameter replaced by the CSM (or the CDM) does not lead to a permissible joint distribution for the full ranges of all parameters, as the Fréchet bounds can be violated.^4^ This puts constraints on the parameters of the joint bivariate distribution which are difficult to translate to the regression parameters when introducing dependency of the parameters on risk factors and other covariates. Moreover, the constraints hinder fitting the models, leading to computational issues such as convergence problems. The objective of this paper is to solve this non-permissibility problem, and to introduce an alternative parameterization guaranteeing a permissible distribution for all combination of values. The alternative parameterization, no longer including the marginal success probabilities, is shown to be of interest on its own, and offers additional insights for particular applications.

In the next section, three settings with illustrative datasets are introduced. Then the new measures and the new parameterization are presented and covariate models for the different parameters and maximum likelihood inference is briefly described. The illustrative datasets are analyzed using the new parameterization and the paper ends with final conclusions, considerations and ideas about further research.

## 2 Applications and datasets

In the following sections, three different settings and specific data examples are introduced in the field of disease control and prevention.

### 2.1 HIV serodiscordance among couples in Mozambique

We consider the same setting as in Juga et al.,^3^ based on data from the 2009 National Survey of Prevalence, Risk Behavioural and Information about HIV and AIDS (INSIDA^5^). This survey was a cross-sectional two-stage survey, carried out by the National Institute of Health in collaboration with the National Bureau of Statistics of Mozambique. The objective is to model HIV serodiscordance among couples as a function of different risk factors and other covariates.

Let $y_{\mathrm{ij}}=(y_{\mathrm{ij}1},y_{\mathrm{ij}2})$ denote the HIV status (1 if positive, 0 if negative) of a (female, male)-couple $j=1,\ldots ,n_{i}$ in enumeration area (EA) *i* with *n_i_* sampled couples, i=1,…,N. For the INSIDA data, the total number of EAs is *N* = 270 and $\sum_{i=1}^{N}n_{i}=2159$ is the total number of couples. Expressing that the covariates $x_{1,\mathrm{ij}},x_{2,\mathrm{ij}}$ and $x_{3,\mathrm{ij}}$ can be possibly different subvectors of the full covariate/factor vector *x_ij_*, Juga et al.^3^ fitted several joint models for both marginal probabilities to be HIV positive, complemented with a new conditional (sero)discordance measure CDM (defined in Section 3):

(1) $$\begin{array}{c} \text{logit}(P\{y_{\mathrm{ij}1}=1\})=\beta_{1}^{T}x_{1,\mathrm{ij}}+b_{1,i},\\ \text{logit}(P\{y_{\mathrm{ij}2}=1\})=\beta_{2}^{T}x_{2,\mathrm{ij}}+b_{2,i},\\ \text{logit}(\text{CDM})=\beta_{3}^{T}x_{3,\mathrm{ij}}+b_{3,i} \end{array}$$

where

(2) $$\left(b_{1,i},b_{2,i},b_{3,i})\;∼N_{3}(0,\Sigma \right)$$

are distributed as a trivariate normal distribution with mean zero-vector and covariance matrix Σ. Note that $x_{\ell ,\mathrm{ij}}$ (ℓ=1,2,3) are vectors of covariates, and some of these covariates are specific for the male/female individual, some specific for the couple, and some are at the level of the province. They considered different choices for the covariance matrix Σ=VRV (including full/partial correlated, full/partial shared, full/partial equal, independent). From their final model, Juga et al.^3^ concluded that the HIV prevalence for the province where a couple was located as well as the union number for the woman within a couple is a factor associated with HIV serodiscordance.

As will be discussed in Section 3, the parameterization with both HIV marginal probabilities and the conditional discordance measure CDM does not satisfy the Fréchet inequalities,^4^ causing computational difficulties with some of the models. In Section 5.1 we will reanalyze these data with the same type of models, but based on our new parameterization as introduced in Section 3.2.

### 2.2 Varicella Zoster Virus and Parvo B19 concordance

The Varicella-Zoster Virus (VZV) and the Parvo B19-virus (B19) are similar in that transmission occurs during close contacts. The contact rate and the infectiousness of the pathogen determine the spread of the infection in a population. It has been shown that the contact rate depends on age through heterogeneity in mixing of individuals from different age-classes. Several approaches have been proposed to model multi-sera data. Hens et al.^6^ used a marginal model (bivariate Dale model^7^ with odds ratio as association parameter) and conditional models (modelling one infection status conditional on the other) to model the multi-sera VZV-B19 data from Belgium. Hens et al.^8^ studied the behaviour of the bivariate-correlated gamma frailty model for cross-sectionally collected serological data on Hepatitis A and B.

Here we reanalyze the Belgian VZV-B19 serological data. In a period from November 2001 until March 2003, 2381 serum samples in Belgium were collected and consecutively tested for VZV and B19.^9^ Together with the test result for VZV and B19, gender and age of the individuals were recorded. Samples from children under 6 months were omitted, as test results are driven by maternal antibodies in this early stage of life. The maximum age of 40 was fixed by design; it was considered not important to test for older ages given that it concerns childhood infections.

**Figure 1.**
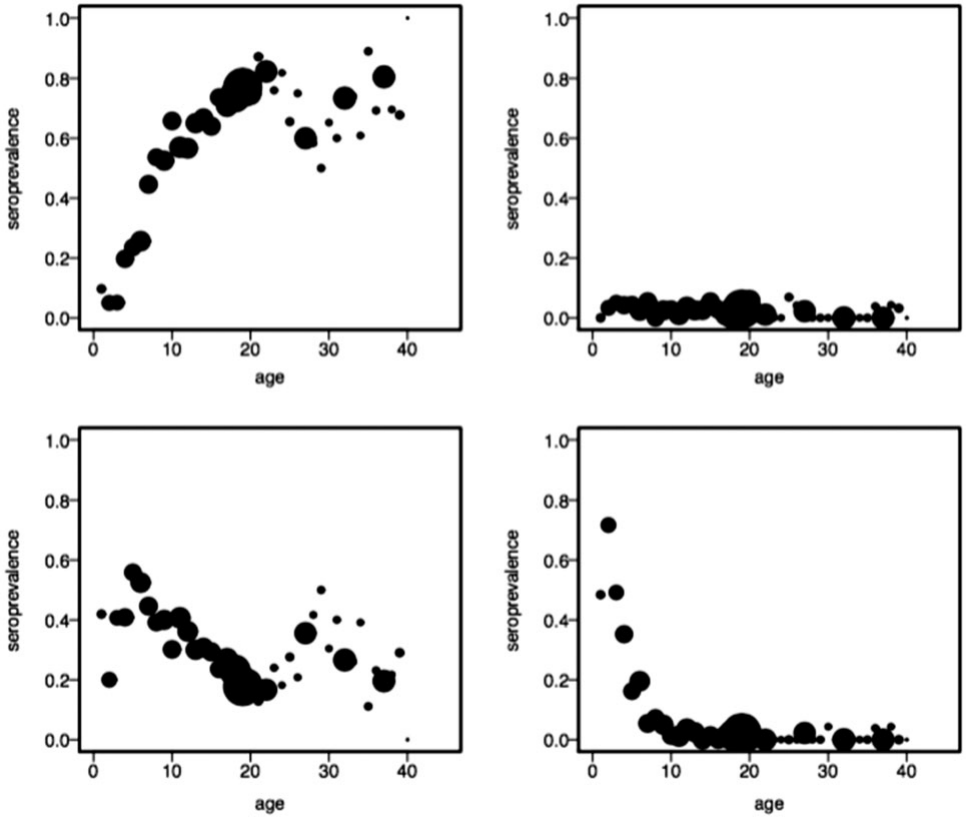
VZV and B19 data, as function of age. Proportion of samples that tested positive on both VZV and B19 (top left panel), that tested positive on B19 only (top right panel), that tested positive on VZV only (lower left panel), and that tested negative on both viruses (lower right panel), based on a cross-sectional survey in Belgium anno 2001–2003. The size of the dots is proportional to the number of serum samples collected in the corresponding age category.

Figure 1 depicts the bivarate distribution of VZV and B19 as a function of age. In Section 5.2, we will propose and discuss new measures to provide other and new insights in the joint occurrence of both infections, as function of age and gender.

### 2.3 Diagnostic performance and concordance of the Whooley questions

Based on a cross-sectional validation study, conducted with 766 patients aged ≥75 from UK primary care and recruited via 17 general practices based in the North of England during the pilot phase of a randomized controlled trial, Bosanquet et al.^10^ assessed the diagnostic performance of the Whooley questions (Whooley et al.^11^) as a screening tool for major depression disorder (MDD) amongst older adults in UK primary care. Sensitivity, specificity, and likelihood ratios comparing the index test (two Whooley questions) for an MDD-diagnosis were ascertained by the reference standard Mini International Neuropsychiatric Interview (MINI^12^). Participants completed a self-reported, written version of the index test, the Whooley questions: (WQ1) During the past month, have you often been bothered by feeling down, depressed, or hopeless? (yes = 1/no = 0); (WQ2) During the past month, have you often been bothered by little interest or pleasure in doing things? (yes = 1/no = 0). In the standard method of scoring the Whooley questions, participants who respond yes to at least one of the two questions were classified as screening positive for depression.

Table 1 shows a 2 × 2 table cross-classifying the index test (positive if being positive for at least one Whooley question) with MINI as the golden standard reference (GSR), as well as tables cross-classifying both Whooley questions against each other, unconditionally and conditional on the GSR status. Concordance between index and golden standard reference, and concordance and discordance between both Whooley questions are of interest and will be discussed in Section 5.3.

**Table 1.** Whooley questions data.

	Index Test	
GSR	0	1
0	458	273
1	2	33
				WQ2		
	WQ2		GSR = 0		GSR = 1	
WQ1	0	1	0	1	0	1
0	460	41	458	40	2	1
1	95	170	91	142	4	28

Left table: index test versus golden standard reference. Right table: Whooley Question 2 versus Whooley Question 1, unconditionally and conditional on the golden standard reference.

## 3 Measuring con(dis)cordance and (a)synchrony

First, we briefly review existing measures, including the conditional synchrony measure. Next the new parameterization is introduced, discussed and relations with other parameters are examined. A final section focuses on the estimation of the new parameters by maximum likelihood.

### 3.1 Existing measures

Consider a bivariate binary outcome $y=(y_{1},y_{2})$ and a (possibly multivariate) covariate *x* and let

(3) $$\pi_{k\ell }(x)=P(y_{1}=k,y_{2}=\ell |x),k,\ell =0,1$$

where $0<\pi_{k\ell }(x)<1$ and $\sum_{k,l=0}^{1}\pi_{k\ell }(x)=1$, denote the conditional joint distribution of *y* given *x* (dependency of *x* suppressed from notation, if not relevant), with marginal conditional (success) probabilities $\pi_{1+}(x)=\pi_{10}(x)+\pi_{11}(x)$ and $\pi_{+1}(x)=\pi_{01}(x)+\pi_{11}(x)$. Many parameterizations are theoretically possible, but in many cases interest goes in the effect of *x* on the marginal probabilities (most often with a logit link allowing an odds ratio interpretation), complemented with an association parameter, such as the correlation $\rho =(\pi_{11}-\pi_{1+}\pi_{+1})/\sqrt{\pi_{1+}(1-\pi_{1+})\pi_{+1}(1-\pi_{+1})}$, (with a Fisher-z link) or the odds ratio $\varphi =(\pi_{00}\pi_{11})/(\pi_{01}\pi_{10})$ (with a log link).

But such association measures are not the target parameter of interest in case interest goes to con(dis)cordance or (a)synchrony. In the context of measuring synchrony in neuronal firing, Faes et al.^2^ stated that the odds ratio is less suitable to quantify synchrony due to its symmetry, treating 0–0 matches of equal importance as 1–1 matches, and proposed a new measure of synchrony, the conditional synchrony measure CSM, defined as

(4) $$\text{B}\mathrm{CSM}(x)=\frac{\pi_{11}(x)}{\pi_{10}(x)+\pi_{01}(x)+\pi_{11}(x)}$$

being the probability of two neurons firing together, given that at least one of the two is active. In order to model HIV serodiscordance among couples in Mozambique, Juga et al.^3^ introduced the conditional (sero)discordance measure BCDM(x)=1-BCSM(x) and showed that the CDM measure is a more direct and relevant measure to study the effects of risk factors.

A limitation of this parameterization, with the marginal probabilities combined with BCSM(x) or BCDM(x), is that not all values of $\pi_{1+}(x),\pi_{+1}(x)$ and of BCSM(x) (or BCDM(x)) result in a permissible joint distribution $\pi_{k\ell }(x)$. Indeed, the Fréchet bounds^4^ need to hold: given values $0<\pi_{1+}(x)<1$ and $0<\pi_{+1}(x)<1$, a permissible joint distribution is only obtained if

(5) $$\max\{1-\frac{\pi_{1+}(x)}{\pi_{+1}(x)},1-\frac{\pi_{+1}(x)}{\pi_{1+}(x)}\}\leq \text{B}\mathrm{CDM}(x)\leq \min\{\pi_{1+}(x)+\pi_{+1}(x),1\}$$

When modelling the dependency on *x* and possibly including additional random effect structures on all three parameters ($\pi_{+1},\pi_{1+}$ and BCDM or BCSM) to account for additional heterogeneity, the constraints (equation (5)) may cause problems when fitting some models (computational issues, non-convergence,…).

### 3.2 New measures and new parameterization

While it is common to include the two marginal probabilities as part of a model parameterization, particular alternative parameters might be of interest too and might shed more light on the research questions at hand. In the three applications of interest, the focus is not on the marginal probabilities. The new parameterization proposed here abandons the common starting point of adding the parameter of interest to the marginal probabilities, but takes an opposite approach: next to the CSM or CDM measure, which other parameters of interest can be introduced to obtain a complete parameterization of the joint distribution?

As a first parameter, define the conditional probability that *y*_1_ is positive, given that both disagree

(6) $$\pi =P(y_{1}=1|y_{1}\neg =y_{2})=\frac{\pi_{10}}{\pi_{10}+\pi_{01}}$$

or, alternatively, 1-π being the probability that *y*_2_ is positive in a disagreeing pair (*y*_1_, *y*_2_). This parameter focuses on disagreeing pairs and the probability that one is more dominant than the other.

Note that π=0.5 implies symmetry ($\pi_{k\ell }=\pi_{\ell k}$) and hence marginal homogeneity ($\pi_{1+}=\pi_{+1}$), and π>0.5 implies that $\pi_{1+}>\pi_{+1}$. Actually *π* is the central parameter in McNemar’s (exact) test for matched pairs with, under the null hypothesis of marginal homogeneity, π^∼Bin(n*,0.5) with n* the total number of disagreeing pairs.^1^ This implies that, although the two marginal success probabilities $\pi_{1+},\pi_{+1}$ are no longer model parameters, their equality $\pi_{1+}=\pi_{+1}$ can still be directly tested, while accounting for covariate effects. Actually, instead of the parameter *π*, one could use the relative difference

$$2\pi -1=\frac{\pi_{10}-\pi_{01}}{\pi_{10}+\pi_{01}}=\frac{\pi_{1+}-\pi_{+1}}{\pi_{10}+\pi_{01}}$$

In the sequel, we will use definition (6) and refer to it as the (marginal) homogeneity parameter.

As a second parameter, define (as before) the “positive” conditional synchrony measure CSM, being the probability that both are agreeing (both positive) given that at least one is positive

(7) $$\sigma_{+}=P(y_{1}=y_{2}|y_{1}+y_{2}\geq 1)=\frac{\pi_{11}}{\pi_{10}+\pi_{01}+\pi_{11}}$$

or, alternatively, the (positive) CDM, now denoted as $\delta_{+}=1-\sigma_{+}$.

Finally, define the third parameter as the “negative” conditional synchrony measure, being the probability that both are agreeing (both negative), given that at most one is positive

(8) $$\sigma_{-}=P(y_{1}=y_{2}|y_{1}+y_{2}\leq 1)=\frac{\pi_{00}}{\pi_{00}+\pi_{10}+\pi_{01}}$$

Again, the third parameter can also be defined as (negative) CDM, being $\delta_{-}=1-\sigma_{-}$.

The homogeneity parameter *π* determines the relative ratio of the off-diagonal probabilities of disagreement, independently of the values of $\sigma_{+}$ and $\sigma_{-}$, whereas the parameters $\sigma_{+},\sigma_{-}$ (or alternatively $\delta_{+},\delta_{-}$) focus on the diagonal probabilities of agreement, and

$$\pi_{11}/\pi_{00}=(\frac{\sigma_{+}}{1-\sigma_{+}})/(\frac{\sigma_{-}}{1-\sigma_{-}})$$

The measure $\sigma_{+}$ tends to 1 if and only if $\sigma_{-}$ does so. The parameters $\delta_{+},\delta_{-},\sigma_{+},\sigma_{-}$ are invariant for switching *y*_1_ with *y*_2_, but *π* will switch to 1-π. Depending on the application and the particular parameters of interest, one can opt for a particular combination, e.g. $\pi ,\delta_{+}$ and $\sigma_{-}$. The joint probabilities $\pi_{k\ell }$ can easily be expressed in terms of the new parameters, e.g. in terms of $\pi ,\sigma_{+}$ and $\sigma_{-}$

$$\begin{array}{c} \pi_{00}=\frac{\sigma_{-}(1-\sigma_{+})}{1-\sigma_{-}\sigma_{+}},\pi_{10}=\frac{\left(1-\sigma_{-})(1-\sigma_{+}\right)}{1-\sigma_{-}\sigma_{+}}\pi ,\\ \pi_{01}=\frac{\left(1-\sigma_{-})(1-\sigma_{+}\right)}{1-\sigma_{-}\sigma_{+}}(1-\pi ),\pi_{11}=\frac{\sigma_{+}(1-\sigma_{-})}{1-\sigma_{-}\sigma_{+}} \end{array}$$

It can also readily be shown that, for any combination of values for the three conditional probabilities $0<\pi ,\sigma_{-},\sigma_{+}<1$, the Fréchet bounds are satisfied, and we obtain a permissible joint distribution $\pi_{k\ell }$.

The marginal success probabilities can be written as

$$\pi_{1+}=\frac{\left(1-\sigma_{-})(1-(1-\sigma_{+})(1-\pi )\right)}{1-\sigma_{-}\sigma_{+}},\pi_{+1}=\frac{\left(1-\sigma_{-})(1-(1-\sigma_{+})\pi \right)}{1-\sigma_{-}\sigma_{+}}$$

and the odds ratio as

(9) $$\varphi =(\frac{\sigma_{-}}{1-\sigma_{-}})(\frac{\sigma_{+}}{1-\sigma_{+}})\frac{1}{\pi (1-\pi )}$$

Identity (9) shows that the odds ratio φ decomposes in three factors, each related to one of the three new parameters. The association in terms of the odds ratio increases multiplicatively with the odds of both synchrony measures $\sigma_{-}$ and $\sigma_{+}$, and converges to infinity as the homogeneity parameter *π* tends to 0 or 1. The minimal value of φ, for fixed values of $\sigma_{-}$ and $\sigma_{+}$, is obtained for π=0.5, corresponding to marginal homogeneity. Of course, this factorization is not helping in characterizing independence, but independence is not of interest in our settings of interest.

The relation with Cohen’s kappa measure of agreement takes the form

$$\kappa =\frac{\sigma_{-}(1-\sigma_{+})+\sigma_{+}(1-\sigma_{-})-K(\pi ,\sigma_{-},\sigma_{+})}{\left(1-\sigma_{-}\sigma_{+})-K(\pi ,\sigma_{-},\sigma_{+}\right)}$$

for a rather complicated function $K(\pi ,\sigma_{-},\sigma_{+})$. But an immediate consequence is that there is perfect agreement according to Cohen’s kappa, *κ* = 1, if and only if there is perfect negative ($\sigma_{-}=1$) or perfect positive ($\sigma_{+}=1$) conditional synchrony.

A bit different and more “asymmetric setting” is that of measuring the accuracy of diagnostic tests. Assume *y*_1_ represents the true disease status, and *y*_2_ another alternative test. Sensitivity $\text{Se}=P(y_{2}=1|y_{1}=1)$ and specificity $\text{Sp}=P(y_{2}=0|y_{1}=0)$ relate to the new parameters as

$$\text{Se}=\frac{\sigma_{+}}{\sigma_{+}+(1-\sigma_{+})\pi },\text{Sp}=\frac{\sigma_{-}}{\sigma_{-}+(1-\sigma_{-})(1-\pi )}$$

and for the positive predictive value $\text{PPV}=P(y_{1}=1|y_{2}=1)$ and the negative predictive value $\text{NPV}=P(y_{1}=0|y_{2}=0)$ it holds that

$$\text{PPV}=\frac{\sigma_{+}}{\sigma_{+}+(1-\sigma_{+})(1-\pi )},\text{NPV}=\frac{\sigma_{-}}{\sigma_{-}+(1-\sigma_{-})\pi }$$

First of all, note that Se and PPV do not depend on $\sigma_{-}$ and Sp and NPV do not on $\sigma_{+}$. As to be expected, Se and NPV decrease whereas Sp and PPV increase with the homogeneity parameter *π*. If *π* is very close to 1, Se$\approx \sigma_{+}$ and Sp≈1. Furthermore, in that case, PPV≈1, and NPV$\approx \sigma_{-}$. Similarly, if *π* is very close to 0, Se≈NPV≈1, Sp$\approx \sigma_{-}$ and PPV$\approx \sigma_{+}$. Se and PPV increase with $\sigma_{+}$ and Sp and NPV increase with $\sigma_{-}$. All of them tend to 1 whenever $\sigma_{+}$ tends to 1 (and thus $\sigma_{-}$ tends to 1).

The diagnostic odds ratio

(10) $$\text{DOR}=(\frac{\text{Se}}{1-\text{Se}})(\frac{\text{Sp}}{1-\text{Sp}})=(\frac{\text{PPV}}{1-\text{PPV}})(\frac{\text{NPV}}{1-\text{NPV}})=(\frac{\sigma_{+}}{1-\sigma_{+}})(\frac{\sigma_{-}}{1-\sigma_{-}})\frac{1}{\pi (1-\pi )}$$

is used as a measure of the effectiveness of a diagnostic or screening test. It is independent of prevalence, and it is a single indicator of test performance, ranging from zero to infinity and higher values (above 1) are indicative of better test performance.^13^ Equation (10) decomposes the DOR in three factors: given the value of the marginal homogeneity parameter *π*, higher values of the DOR correspond to higher values of one or both synchrony measures $\sigma_{+},\sigma_{-}$.

Although our application as introduced in Section 2.3 is based on a cross-sectional study, allowing to estimate the disease prevalence by the case study prevalence, this might be not the case for other study designs. Consider for instance the case-control design, with data about a screening test result for the diseased and non-diseased subpopulation (as defined by a golden standard or reference test). Such a design allows the estimation of the sensitivity and specificity, but not the prevalence of the disease. The formulas

$$\begin{array}{c} \pi =\frac{(1-\text{Se})\text{Prev}}{\left(1-\text{Se})\text{Prev}+(1-\text{Sp})(1-\text{Prev}\right)},\\ \sigma_{+}=\frac{\text{Se}\times \text{Prev}}{1-\text{Sp}(1-\text{Prev})},\\ \sigma_{-}=\frac{\text{Sp}(1-\text{Prev})}{1-\text{Se}\times \text{Prev}} \end{array}$$

with $\text{Prev}=\pi_{1+}$ the prevalence of the disease, show the dependency of the homogeneity parameter and both conditional synchrony measures on the disease prevalence. These formulas allow us to combine data with knowledge about the disease prevalence (from other data or literature) to estimate or to model the parameters *π*, $\sigma_{+}$ and $\sigma_{-}$ for a case-control study (typically in the Bayesian paradigm).

## 4 Estimation and inference

Consider quadrinomial observations $y_{\mathrm{jk}\ell }=I(y_{j1}=k)I(y_{j2}=\ell )$ for *k* = 0, 1 and ℓ=0,1, for j=1,…,n. Then, by the orthogonality $\pi ⊥(\sigma_{-},\sigma_{+})$, the quadrinomial likelihood, with $\pi_{00}+\pi_{01}+\pi_{10}+\pi_{11}=1$

$$L_{\left(4\right)}(\pi_{00},\pi_{01},\pi_{10},\pi_{11})=\overset{n}{\underset{j=1}{\Pi }}\pi_{00}^{y_{j00}}\pi_{01}^{y_{j01}}\pi_{10}^{y_{j10}}\pi_{11}^{y_{j11}}$$

factorizes into a trinomial and binomial likelihood $L_{\left(4\right)}=L_{\left(3\right)}\times L_{\left(2\right)}$ with

$$\begin{array}{c} L_{\left(3\right)}(\sigma_{-},\sigma_{+})=\overset{n}{\underset{j=1}{\Pi }}\pi_{00}^{y_{j00}}\pi_{11}^{y_{j11}}(\pi_{01}+\pi_{10}{)}^{y_{j01}+y_{j10}},\\ =\frac{\left(1-\sigma_{-})(1-\sigma_{+}\right)}{\left(1-\sigma_{-}\sigma_{+}\right)}\overset{n}{\underset{j=1}{\Pi }}(\frac{\sigma_{-}}{1-\sigma_{-}}{)}^{y_{j00}}(\frac{\sigma_{+}}{1-\sigma_{+}}{)}^{y_{j11}} \end{array}$$

with

$$L_{\left(2\right)}(\pi )=\overset{n}{\underset{j=1}{\Pi }}\pi^{y_{j10}}(1-\pi {)}^{y_{j01}}$$

In case no parameters are common to the models for $\left(\sigma_{-},\sigma_{+}\right)$ and *π*, both likelihoods can be maximized separately, and, in case interest only goes to the conditional synchrony measures, the disagreeing observations can be collapsed and it suffices to only maximize the trinomial likelihood.

The dependency of the three conditional probabilities $\pi ,\sigma_{+}$ and $\sigma_{-}$ (or any other eligible combination of interest from the sets $\left\{\sigma_{+},\delta_{+}\right\}$ and $\left\{\sigma_{-},\delta_{-}\right\}$) on covariates can be modelled with three components

(11) $$h_{1}(\pi_{j})=\beta_{1}^{T}x_{1j},h_{2}(\sigma_{+j})=\beta_{2}^{T}x_{2j},h_{3}(\sigma_{-j})=\beta_{3}^{T}x_{3j}$$

where *h*_1_, *h*_2_ and *h*_3_ are link functions (logit, probit, cloglog,…). We will focus on the logit link as it allows a more appealing interpretation of covariate effects in terms of odds ratios. The model components (11) can be embedded in different frameworks of estimation and inference; we will opt for full maximum likelihood.

Note that, when using the relative difference parameter 2π-1, ranging from −1 to 1, the logit link is not appropriate but rather a Fisher-z link would be in order. The identity, with $\Delta_{r}=2\pi -1$

$$\text{log}(\frac{\pi }{1-\pi })=-\text{log}(\frac{1-\pi }{\pi })=\text{log}(\frac{1+\Delta_{r}}{1-\Delta_{r}})$$

implies that models for *π*, 1-π and Δ*_r_* with respective links are identical (only opposite slopes for the models for 1-π). Depending on the application at hand, one might be more interested in interpreting the estimates in terms of *π*, 1-π or Δ*_r_*. For the latter choice, a zero intercept would reflect marginal homogeneity for all covariate values equal to 0, and the effect of a covariate as represented by the estimated slope would reflect non-homogeneity in one or the other direction: a positive slope would indicate a higher marginal probability of success in the first variable, and a negative slope would indicate a higher marginal probability of success in the second variable.

## 5 Applications

In this section we revisit the three applications introduced in Section 2 and show how in each example model (11) can be formulated and we illustrate the use and interpretation of the three conditional probabilities $\pi ,\sigma_{+}$ and $\sigma_{-}$ (or variations thereof). Data analyses were performed in SAS version 9.4 using PROC NLMIXED (exemplifying code in the supplemental material).

### 5.1 Modelling HIV serodiscordance among couples in Mozambique

We reanalyse the HIV data introduced in Section 2.1 based on model components for i) the (homogeneity) probability *π_ij_* that the female partner of couple *j* in EA *i* is HIV positive, given that both partners differ in their HIV status; ii) the probability $\delta_{+\mathrm{ij}}$ that only one is HIV positive, given that at least one of the two partners is positive (positive serodiscordance); and iii) the probability $\sigma_{-\mathrm{ij}}$ that both are negative given that at most one of the two partners is positive (negative seroconcordance)

$$\begin{array}{c} \text{logit}(\pi_{\mathrm{ij}})=\beta_{1}^{T}x_{1,\mathrm{ij}}+b_{\pi ,i}\\ \text{logit}(\delta_{+\mathrm{ij}})=\beta_{2}^{T}x_{2,\mathrm{ij}}+b_{\delta_{+},i},\\ \text{logit}(\sigma_{-\mathrm{ij}})=\beta_{3}^{T}x_{3,\mathrm{ij}}+b_{\sigma_{-},i} \end{array}$$

with

(12) $$\left(b_{\pi ,i},b_{\delta_{+},i},b_{\sigma_{-},i})∼N_{3}(0,\Sigma \right)$$

trivariate normally distributed random EA-effects, with mean zero-vector and covariance matrix Σ.

Most of the sample designs for household surveys such as INSIDA are complex and involve stratification, multistage sampling, and unequal sampling rates, and it is necessary to account for the particular survey design in the statistical analyses using appropriate weights. We followed the same approach as Juga et al.^3^ For more details on the calculation of the weights as used in our analyses, we refer to Juga et al.^3^

After following the same model building procedure as in Juga et al., the best fitting final model had no random EA-effect $b_{\pi ,i}$ on parameter *π_ij_* and correlated random EA-effects $\left(b_{\delta_{+},i},b_{\sigma_{-},i}\right)$ on $\left(\delta_{+\mathrm{ij}},\sigma_{-\mathrm{ij}}\right)$. This model has an AIC value of 2509.2, considerably improving the fit of the best model in Juga et al.^3^ (AIC = 2571.8 for a partial-equal random effects type of model). All models converged, in contrast to the experiences of Juga et al.,^3^ who reported that the models with independent random effects and full or partial random effects did not convergence.

Table 2 shows the estimates of the final model. The following covariates appear in the final model with fixed effects: ‘HIV prevalence’ is the prevalence of HIV at the couple’s residence at the level of the province, categorized into three categories using cutpoints of 5% and 15% and with 0–5% as reference category; ‘Union number woman’ refers to whether the woman has been married or lived with a man once (reference category) or more than once; ‘STI man’ is yes if the man answered yes to any of three questions about symptoms of sexually transmitted infections STIs (no is reference); ‘Condom use woman’ refers to whether the female respondent of the couple used a condom the last time she had sexual intercourse with the other partner (yes is reference); ‘Wealth index’ refers to the economic status of the couple with three categories Poorer, Middle and Richer (reference). For more details, see Fishel et al.^14^

**Table 2.** HIV serodiscordance example.

Effect	π	$\delta_{+}$	$\sigma_{-}$
Intercept	−0.82(0.40)^a^	2.48(0.69)^a^	3.65(0.37)^a^
HIV prevalence			
5–15%	–	−0.93(0.53)	−0.88(0.28)^a^
>15%	–	−1.09(0.56)	−1.64(0.32)^a^
Union number woman			
More than once	0.57(0.28)^a^	−0.74(0.26)^a^	−0.49(0.17)^a^
STI man			
Yes	–	−1.41(0.42)^a^	–
Condom use woman			
Not used	1.87(0.79)^a^	–	–
Wealth index			
Poorer	–	1.13(0.37)^a^	0.42(0.22)
Middle	–	0.20(0.37)	0.52(0.24)^a^
Variance components			
$\sigma_{\delta_{+}}^{2}$	–	1.29(0.43)^b^	
$\sigma_{\sigma_{-}}^{2}$	–	0.72(0.20)^b^	
$\rho_{\delta_{+}\sigma_{-}}$	–	−0.62(0.14)^a^	

Note: Estimates (standard errors) of the final model with no random EA-effect for parameter *π_ij_* and correlated random EA-effects $\left(b_{\delta_{+},i},b_{\sigma_{-},i}\right)$ on $\left(\delta_{+\mathrm{ij}},\sigma_{-\mathrm{ij}}\right)$, with variance components $\sigma_{\delta_{+}}^{2},\sigma_{\sigma_{-}}^{2}$ and correlation $\rho_{\delta_{+}\sigma_{-}}$. –A– sign in a column refers to a non-significant effect at 5% after which the covariate was deleted (stepwise).

a Significant at 5% level based on a likelihood ratio test.

b Significant at 5% level, using $\chi_{0,1}^{2}$-mixture.

Comparing our results with those of Juga et al.^3^ and focusing on the common conditional serodiscordance parameter CDM = $\delta_{+}$ in both models, similar effects were obtained for the effect of ‘HIV prevalence’ and ‘Union number woman’. Additional to those effects, our new model identified a significant effect of ‘STI man’ and ‘Wealth index’: the probability for both partners of a couple to differ in HIV status, given that at least one of both is positive, decreases in case the man has reported STIs and increases in case the couple’s wealth index is poorer rather than richer.

The negative synchrony depends on the ‘HIV prevalence’ (the higher the prevalence the lower the synchrony), the ‘Union number woman’ (lower synchrony in case the woman has been married or lived with a man more than once) and the ‘Wealth index’ (higher synchrony for the middle category).

Finally for the homogeneity parameter *π*, it can be observed that marginal homogeneity (both partners having the same probability to be HIV positive) does not hold in case the woman has been married or lived with a man only once and the man has indicated to have no STI symptoms (95% CI [0.166, 0.491] for *π*, implying the probability to be HIV positive is lower for the woman) and in case the woman has been married or lived with a man more than once and the man has indicated to have STI symptoms (95% CI [0.517, 0.960] for *π*, implying the probability to be HIV positive is higher for the woman).

### 5.2 Varicella Zoster Virus and Parvo B19 concordance

Let *y*_1_ refer to the B19 infection status and *y*_2_ to the VZV infection status of the same individual. We are interested in the dependency of *π*, $\sigma_{+}$ and $\sigma_{-}$ on age and gender. Table 3 shows the observed bivariate frequencies of (*y*_1_, *y*_2_), unconditionally and conditionally on gender. Model (11) is applied with an indicator for gender and a cubic spline for age restricted to be linear before the first knot and after the last knot, with knots located at the 9 deciles of the observed age distribution. Gender was not significant for any parameter (*p*-values 0.8762, 0.2002, 0.8641 for *π*, $\sigma_{+}$ and $\sigma_{-}$ respectively). Age has a significant effect on all three parameters $\pi ,\sigma_{+}$ and $\sigma_{-}$ (*p*-values 0.004, <0.0001, <0.0001, respectively).

**Table 3.** VZV and B19 data.

			VZV			
	VZV		Female		Male	
B19	0	1	0	1	0	1
0	174	725	91	357	83	368
1	57	1425	28	733	29	692

Note: B19 versus VZV, unconditionally and conditional on gender.

**Figure 2.**
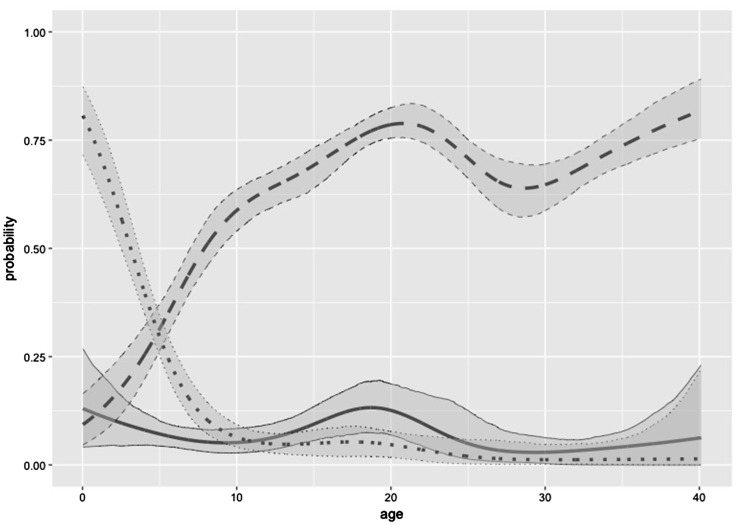
VZV and B19 example. Plot of the fitted *π* (solid line), $\sigma_{+}$ (dashed line) and $\sigma_{-}$ (dotted line) as function of age and based on the final model, together with 95% bootstrap percentile intervals using 1000 nonparametric bootstrap samples.

Figure 2 visualizes the dependencies on age. For an individual who is positive for one and negative for the other virus, the probability is lowest that he/she is positive for B19 (about 0.10) across all ages. Marginal homogeneity clearly does not hold, for any age (*p* < 0.00001). If an individual is positive for at least one virus, the probability $\sigma_{+}$ that he/she is positive for both increases from 0.10 to about 0.80 with a strange bump at the age of 20. The negative conditional synchrony $\sigma_{-}$, being the probability he/she is negative for both given that he/she is at most positive for one of the virus, decreases rapidly during the first 10 years of life, from about 0.80 to about 0.05. The bump around the age of 20, visible more or less for all curves, is caused by an “artefact” in the data, in the sense that the prevalence to be positive for one only, or for both is expected to be monotone as a function of age (the older you are the higher the probability of ever been infected by one of the diseases). Already Figure 1 shows that this expected monotonicity constraint is violated by the patterns shown in the data. This phenomenon and possible model modifications and extensions (covering, e.g., waning immunity) have been presented and discussed in, for example, Abrams and Hens.^15^

Using a bivariate Dale model and splines for the effect of age, Hens et al.^6^ could not reject the null hypothesis of a constant OR (*p* = 0.37). The estimated age and gender independent OR equaled 2.11 with 95% confidence interval (1.45, 3.23). This is another example showing that measures for agreement and for dependency can behave quite differently.

### 5.3 Diagnostic performance and concordance of the Whooley questions

Let *y*_1_ refer to the GSR and *y*_2_ to the index test of the same individual. Bosanquet et al.^10^ report a high sensitivity of 0.943 (95% CI [0.808, 0.993]) and a modest specificity of 0.627 (95% CI [0.590, 0.662]) for the index test (at least one Whooley question positive). Table 4 shows estimates for all parameters of interest. Note that the homogeneity parameter π^=0.007 is very small, implying marginal heterogeneity and that, as noticed in general, $\text{Sp}\approx \sigma_{-},\text{PPV}\approx \sigma_{+}$ and Se≈NPV≈1. So, if index and reference test disagree, it is highly unlikely that the reference test is the positive one. If at least one of the index or reference test is positive, it is unlikely that both are positive (probability of 0.107), so low positive synchrony or concordance. If at least one is negative, they are both negative with probability 0.625.

**Table 4.** Whooley questions example.

	Est	se	95% CI
Se	0.943	0.0392	(0.866, 1.000)
Sp	0.627	0.0179	(0.591, 0.662)
PPV	0.108	0.0177	(0.073, 0.143)
NPV	0.996	0.0031	(0.990, 1.002)
*π*	0.007	0.0051	(0.000, 0.017)
$\sigma_{+}$	0.107	0.0176	(0.073, 0.142)
$\sigma_{-}$	0.625	0.0179	(0.591, 0.660)

Note: Table of Index test × GSR: point estimates, standard error estimates and 95% confidence intervals for sensitivity, specificity, positive predictive value, negative predictive value, probability *π*, positive and negative conditional synchrony.

Next it is interesting to get more insight in the (con/dis)cordance between both Whooley questions, defining the index test, and how this depends on the GSR status. So, let *y*_1_ now refer to WQ1 and *y*_2_ to WQ2. Table 5 shows the estimated parameters, unconditionally and conditionally on the GSR status. SAS code for the model with parameters modelled as a function of the GSR status (MINI) is included in the Supplementary material.

**Table 5.** Whooley questions example.

	Est	se	95% CI
*π*	0.699	0.0394	(0.616, 0.770)
$\sigma_{+}$	0.556	0.0284	(0.499, 0.610)
$\sigma_{-}$	0.772	0.0172	(0.736, 0.804)
*π* | GSR = 0	0.695	0.0402	(0.611, 0.768)
$\sigma_{+}$ | GSR = 0	0.520	0.0302	(0.461, 0.580)
$\sigma_{-}$ | GSR = 0	0.778	0.0171	(0.742, 0.809)
*π* | GSR = 1	0.800	0.1789	(0.308, 0.973)
$\sigma_{+}$ | GSR = 1	0.849	0.0624	(0.683, 0.936)
$\sigma_{-}$ | GSR = 1	0.286	0.1707	(0.072, 0.674)

Note: Table of WQ1 × WQ2, unconditionally and conditionally on GSR status: point estimates, standard error estimates and 95% confidence intervals for probability *π*, positive and negative conditional synchrony.

The homogeneity probability *π* does not depend on the GSR status (*p* = 0.6189). As $\pi ⊥(\sigma_{-},\sigma_{+})$, it is not necessary to refit a simplified model with no effect of the GSR status on *π*. Indeed, fitting such model would lead to exactly the same results for $\sigma_{+}$ | GSR = 0 or 1 and $\sigma_{-}$ | GSR = 0 or 1, and the GSR independent estimate for *π* would equal the estimate 0.699 in the collapsed table, being the first line of results in Table 5. So, whether or not the individual is depressed, if the answers on the two WQ’s disagree, the probability is about 70% that WQ1 is answered positively (95% CI being [0.616, 0.770], marginal homogeneity does not hold). So, individuals with disagreeing answers on both Whooley questions tend to have been more often bothered by feeling down, depressed, or hopeless, than bothered by little interest or pleasure in doing things, regardless of their GSR status.

But $\sigma_{+}$ and $\sigma_{-}$ significantly depend on the GSR status, with *p*-values 0.0011 and 0.0103, respectively. The positive synchrony measure estimate σ^+ increases from 0.520 to 0.849 implying that, given that at least one of the WQ’s is answered positively, the probability that both are positively answered increases substantially for individuals suffering from a major depression disorder (according to GSR). On the other hand, the negative synchrony measure estimate σ^- decreases from 0.778 to 0.286 implying that, given that at most one of the WQ’s is answered positively, the probability that both are negatively answered decreases substantially for individuals suffering from a major depression disorder (according to GSR).

Bosanquet et al.^10^ mentioned in the discussion that the use of the two-item version of the Whooley questions, rather than a three-item version, in which the respondent is asked to state whether they would like help for any difficulties reported, is a potential limitation of their study. However, evidence from recent studies using a third help question, does not provide a conclusive answer whether to include or not the third question. It would be interesting to study in more depth the con- and discordance between all three questions in order to come up with an improved index test.

## 6 Conclusions and discussion

As interest goes to modelling a genuine synchrony/concordance measure rather than a typical association measure such as the odds ratio, the joint distribution of matched pairs of binary data need to reparametrized accordingly. In this contribution, a new parameterization solved the existing permissibility issue with the conditional synchrony measure and related limitations of fitting appropriate models. This new parameterization is based on two synchrony measures, a positive and negative synchrony (or alternatively discordance) parameter, combined with a marginal homogeneity parameter, leading without any restrictions on any of these parameters to a permissible joint distribution for the matched binary pairs, thus facilitating the fitting of more flexible and appropriate models.

The usefulness of the new approach has been illustrated in three different areas of application in disease control and prevention. In the first application, the positive serodiscordance was the main parameter of interest, but also the additional negative seroconcordance provides alternative new insights in this field. While the same characteristic (HIV status) is measured for both partners of a couple in the first application, two different characteristics (VZV and B19 infection status) on one and the same individual are available in the second application. The negative and positive synchrony measures provide new information and insights in the joint process of acquiring both diseases having similar transmission routes. In a third, more distinct application, the accuracy of a screening test is to be assessed in relation to the true disease status (or a gold standard). The new synchrony measures allow to investigate the performance of the diagnostic test from another angle, different from but closely related to well-known accuracy measures such as sensitivity, specificity, predictive values, DOR, etc. and future use of these new measures will shed more light on their ultimate value in this particular field of application.

An advantage of the new approach is that, in case interest only goes to both synchrony measures and their models do not share any parameter in common with the model for the marginal homogeneity parameter, the disagreeing observations can be collapsed and the synchrony measures can be modelled by means of a simplified trinomial likelihood. It may be perceived as a disadvantage that the marginal success probabilities (such as the prevalence of one or both diseases) are not directly estimated or modelled as a function of covariates, as in the currently applied parameterizations. On the other hand, the homogeneity parameter still allows to investigate structural differences in both marginal parameters. Moreover, models for the new parameters $\pi ,\sigma_{+},\sigma_{-}$ imply indirect models for the marginal success probabilities using their relation equations.

A first interesting methodological topic for further research is the modification and application of the HIV model of the first example to same-sex couples, as already mentioned by Juga et al.^3^ They suggested two approaches to deal with the exchangeability of both partners of a couple. But the parameterization proposed here offers an interesting third option. Indeed only the marginal homogeneity parameter depends on the order of the partners in a couple and would not be interpretable when using a random order (actually one would expect homogeneity in case of a random order). Being orthogonal to the other parameters, it would not affect the estimation of the synchrony measures.

Another interesting extension is to examine synchrony between three or more outcomes, as for instance three related infections in our second example. As the number of parameters grows exponentially with the outcome-dimension, defining a full set of appropriate homogeneity and synchrony parameters needs careful considerations in view of the application of interest. For the last example, one often includes an inconclusive category, introducing one or both as a trinomial outcome. An extension in that direction poses interesting challenges. Also in the latter setting, one might like to account for an imperfect GSR by correcting for misclassifications.

## Supplemental Material

Supplemental material for Measures for concordance and discordance with applications in disease control and prevention Click here for additional data file. Supplemental material for Measures for concordance and discordance with applications in disease control and prevention by Marc Aerts, Adelino JC Juga and Niel Hens in Statistical Methods in Medical Research
