# Supplementary material for: Measures for concordance and discordance with applications in disease control and prevention
Source: Stat Methods Med Res. 2018 Sep 3;28(10-11):3086–99. doi: 10.1177/0962280218796252 (PMC6923714; doi:10.1177/0962280218796252)
Supplement: Supplemental material for Measures for concordance and discordance with applications in disease control and prevention [file Supplemental_material.pdf]

Supplemental material

```
/* (CON/DIS)CORDANCE BETWEEN BOTH WHOOLEY QUESTIONS AS A FUNCTION OF
                                          THE GOLDEN STANDARD STATUS */
proc nlmixed data=WORK.DEPRESSION;

/* Starting values for intercepts and slopes */
parms b0_1=0 bA_1=0 b0_2=0 bA_2=0 b0_3=0 bA_3=0;

/* The dependency of the model parameters on the GSR status (MINI) */
eta1=b0_1+bA_1*MINI;
AP10=1/(1 + exp(-eta1));
eta2=b0_2+bA_2*MINI;
PCSM = 1/(1 + exp(-eta2));
eta3=b0_3+bA_3*MINI;
NCSM = 1/(1 + exp(-eta3));

/* The joint probabilities as function of the model parameters */
p11 = (PCSM-NCSM*PCSM)/(1-NCSM*PCSM);
p00 = (NCSM-NCSM*PCSM)/(1-NCSM*PCSM);
p10 = AP10*(1-NCSM)*(1-PCSM)/(1-NCSM*PCSM);
p01 = (1-AP10)*(1-NCSM)*(1-PCSM)/(1-NCSM*PCSM);

/* The model parameters for both statuses of the GSR*/
AP10GS0=1/(1 + exp(-b0_1));
PCSMGS0 = 1/(1 + exp(-b0_2));
NCSMGS0 = 1/(1 + exp(-b0_3));
AP10GS1=1/(1 + exp(-b0_1-bA_1));
PCSMGS1 = 1/(1 + exp(-b0_2-bA_2));
NCSMGS1 = 1/(1 + exp(-b0_3-bA_3));

/* Compute log likelihood for bivariate observations */
ll = depression*pleasure*log(p11) + depression*(1-pleasure)*log(p10)
          + (1-depression)*pleasure*log(p01) + (1-depression)*(1-pleasure)*log(p00);
model ll ~ general(ll);
```

```
estimate ’pi given GSR=0’ AP10GS0;
estimate ’sigma+ given GSR=0’ PCSMGS0;
estimate ’sigma- given GSR=0’ NCSMGS0;
estimate ’pi given GSR=1’ AP10GS1;
estimate ’sigma+ given GSR=1’ PCSMGS1;
estimate ’sigma- given GSR=1’ NCSMGS1;
run;
```


https://mc.manuscriptcentral.com/smmr
